# BUILDING BRIDGES BETWEEN RURAL PROVIDERS AND RACIALLY/ETHNICALLY DIVERSE RETIRED FLORIDA FARM WORKERS

**DOI:** 10.1093/geroni/igad104.0518

**Published:** 2023-12-21

**Authors:** Lisa Ann Wiese, Magdalena Tolea, Joanne Pulido

**Affiliations:** Florida Atlantic University, Boca Raton, Florida, United States; University of Miami Miller School of Medicine, Miami, Florida, United States; Palm Beach State College, Lake Worth, Florida, United States

## Abstract

We report results from a consortium-led randomized controlled clinical trial to optimize rural community health through interdisciplinary detection and care (ORCHID), funded by the Florida Department of Health/Moore Alzheimer’s Disease Research Foundation. This project involving providers, nursing students, office staff, community residents and caregivers, targets a health professional shortage area with a 39% poverty rate. Both the control group (56% non-White, n=11) and intervention group ((93% non-White, n=9) received training on screening tools and updated ADRD information using the Florida Department of Health ADRD Resource Guide. The intervention group is participating in an additional four hours of evening online ADRD diagnosis/treatment training geared towards primary care clinicians, available at no-cost through Washington University. Both groups are being offered in-depth cognitive assessments by Adult Gerontological Nurse Practitioners (AGNP) during home visits. Connections to under-utilized local resources are being facilitated by local nursing students. Initial results include that although pre-post total Alzheimer’s Disease Knowledge Scores (Carpenter et al., 2009) correlated strongly (r =.93; p =.003), there was no significant difference in knowledge (p =.07) in the control group. There was also no significant increase in ADRD diagnostic rates, and AGNP visits were not requested. Instead, the control group providers report the routine practice of conducting annual cognitive screening in persons over age 65, and referring patients as needed to the nearest neurology providers 30 miles away, which residents do not visit. However, connections to local resources increased by 65%. New ADRD diagnosis/treatment rates in the intervention group will be available August, 2023.

